# Proteomic characterization of an isolated fraction of synthetic proteasome inhibitor (PSI)-induced inclusions in PC12 cells might offer clues to aggresomes as a cellular defensive response against proteasome inhibition by PSI

**DOI:** 10.1186/1471-2202-11-95

**Published:** 2010-08-12

**Authors:** Xing'an Li, Yingjiu Zhang, Peng Xie, Jinhua Piao, Yihong Hu, Ming Chang, Tao Liu, Linsen Hu

**Affiliations:** 1 Department of Neurology, The First Affiliated Hospital, Jilin University, Changchun 130021, China; 2 Key Laboratory for Molecular Enzymology and Engineering, Ministry of Education (Jilin University), Changchun 130021, China; 3 Department of Neurology, The First Affiliated Hospital, Chongqing Medical University, Chongqing 400016, China; 4 Department of Cardiovascular Pediatrics, The First Affiliated Hospital, Jilin University, Changchun 130021, China; 5 College of Life Sciences, Jilin University, Changchun 130021, China

## Abstract

**Background:**

Cooperation of constituents of the ubiquitin proteasome system (UPS) with chaperone proteins in degrading proteins mediate a wide range of cellular processes, such as synaptic function and neurotransmission, gene transcription, protein trafficking, mitochondrial function and metabolism, antioxidant defence mechanisms, and apoptotic signal transduction. It is supposed that constituents of the UPS and chaperone proteins are recruited into aggresomes where aberrant and potentially cytotoxic proteins may be sequestered in an inactive form.

**Results:**

To determinate the proteomic pattern of synthetic proteasome inhibitor (PSI)-induced inclusions in PC12 cells after proteasome inhibition by PSI, we analyzed a fraction of PSI-induced inclusions. A proteomic feature of the isolated fraction was characterized by identification of fifty six proteins including twenty previously reported protein components of Lewy bodies, twenty eight newly identified proteins and eight unknown proteins. These proteins, most of which were recognized as a profile of proteins within cellular processes mediated by the UPS, a profile of constituents of the UPS and a profile of chaperone proteins, are classed into at least nine accepted categories. In addition, prolyl-4-hydroxylase beta polypeptide, an endoplasmic reticulum member of the protein disulfide isomerase family, was validated in the developmental process of PSI-induced inclusions in the cells.

**Conclusions:**

It is speculated that proteomic characterization of an isolated fraction of PSI-induced inclusions in PC12 cells might offer clues to appearance of aggresomes serving as a cellular defensive response against proteasome inhibition.

## Background

The ubiquitin proteasome system (UPS) involves a serial enzymatic cascade of ubiquitin dependent protein degradation through which to prevent aberrant and potentially cytotoxic proteins from forming insoluble protein aggregates [1]. Chaperones are a group of protein folding catalysts and protein transporters which also emerge as active participants in protein degradation [2]. Cooperation of constituents of the UPS with chaperone proteins mediate a wide range of cellular processes, such as synaptic function and neurotransmission, gene transcription, protein trafficking, mitochondrial function and metabolism, antioxidant defence mechanisms, and apoptotic signal transduction [3-5]. Dysfunction of the UPS has been implicated in protein aggregation as a possible cause for pathogenesis in a number of the age-related neurodegenerative and chronic neurological disorders [6]. Parkinson's disease (PD) viewed as a representative of these disorders is characterized by selective death of dopaminergic neurons in the substantia nigra pars compacta (SNpc) and by appearance of abundant proteinaceous inclusions known as Lewy bodies (LBs) [7].

One of current concepts in PD pathogenesis is to understand the mechanism of LBs. Protein aggregation has been demonstrated to play the role in the developmental stages of LBs at cellular and molecular levels [8,9]. Substantial progress has been made in understanding of the mechanism for LBs, aggresome-related process [10,11]. According to this hypothesis, dysfunction of the UPS contributes to aggregation of aberrant and potentially cytotoxic proteins into insoluble protein aggregates. One protective way cells handle the species of aggregated proteins is to compartmentalize them in specialized inclusions called as aggresomes [12,13]. The aggresome machinery is activated as the last resort if constituents of the UPS and chaperone proteins fail to reverse increasing level of the target proteins through transient up-regulation of gene expression in the stressful environment [14-16]. It is supposed that constituents of the UPS and chaperone proteins are recruited into aggresomes where aberrant and potentially cytotoxic proteins may be sequestered in an inactive form [17,18].

However, proteins in aggresomes are to be revealed if we elaborate on possible causation of formation of LBs: largely owing to dysfunction of the UPS. Currently, the proteomic analysis of proteinaceous inclusions, whether for patients with PD or in vitro models of PD due to disruption of mitochondria, offers a valuable tool to study protein composition of aggresomes [19,20]. Traditionally, cellular fractionation has provided the means to isolate a fraction of LBs from brain tissue of LB variant of Alzheimer's disease patients [21]. Therefore, an isolated fraction of inclusions as the starting material is suitable for proteomic analysis [22]. Although it remained to be determined that LBs are *bona fide *in vivo correlates of aggresomes [13], aggresomes and LBs are similar in protein composition as well as morphological structures [23]. At least, on the premise of the inducible aggresomes in cultured cells [13] and the emphasized purity and yield of organelles isolated through cellular fractionation of samples [22], proteomic characterization of inclusions can advance our knowledge about nature of aggresomes [19].

The aim of this present work was to determinate the proteomic pattern of proteasome inhibitor (PSI)-induced inclusions formed in PC12 cells after proteasome inhibition by PSI. Our main results were that a fraction of PSI-induced inclusions isolated from the cells was qualified for proteomic analysis and that a proteomic feature of the isolated fraction was characterized by the identification of fifty six proteins.

## Results

### Evaluation of cell survival

**Figure 1:**
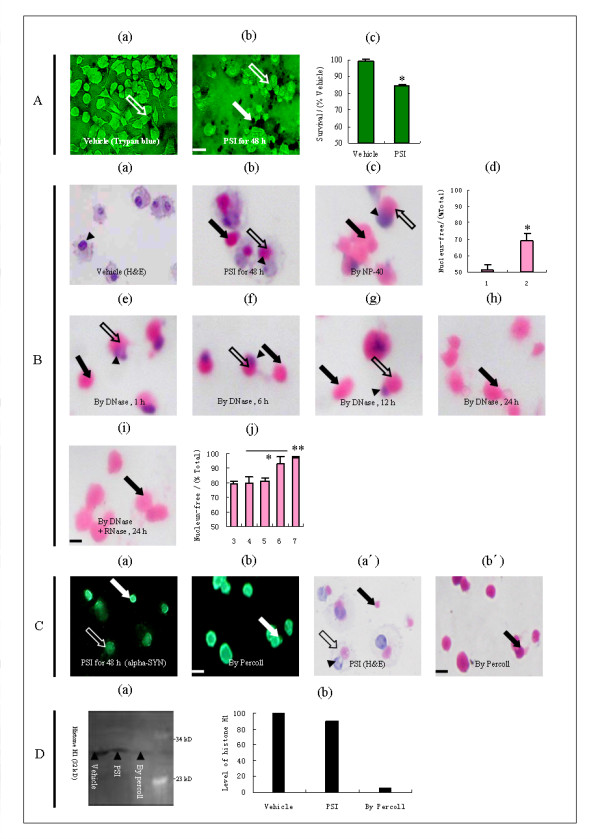
**Effect of proteasome inhibition by PSI on both cell survival and formation of PSI-induced inclusions in PC12 cells, and assessment of a three-process fractionation procedure on isolation of PSI-induced inclusions from the cells**. A, trypan blue staining of cells: a, cell survival (open arrows) alone in cells exposed to DMSO vehicle for 48 h; b, cell survival and addition of cell death (closed arrows) in cells exposed to 10 μM PSI for 48 h; c, percentages of the number of living cells. B, H&E histological staining of inclusions: a, cells in the vehicle; b, nucleus-binding (open arrows) and nucleus-free (closed arrows) inclusions formed in cells in 10 μM PSI for 48 h; c, the two morphological types of inclusions in the fraction of initial pellets; d, percentages of nucleus-free to total inclusions in the cells in PSI (Column 1) and in the fraction of initial pellets (Column 2); e-h, the one (or two) morphological type (s) of inclusions in the first four temporary fractions of resulting pellets; i, the one morphological type of inclusions in the final fraction of resulting pellets; j, percentages of nucleus-free to total inclusions in the five fractions of resulting pellets (Column 3-7). C, alpha-SYN immunostaining for and H&E histological staining of inclusions: a and a', cells in PSI; b and b', an isolated fraction of inclusions. D, immunoblotting for histone H1 in inclusions: a, bands of histone H1 in gel image; b, quantitative level of histone H1 in densitometry. Scale bar, 10 μm.

Using the trypan blue staining, we examined the plasma membrane integrity of the cells following exposure to PSI for 48 h. Compared to the cells in dimethyl sulfoxide (DMSO) vehicle which were only shown as normal living cells excluding trypan blue stain (Figure 1A:a), the cells in PSI were shown as both abnormal living cells and dead cells, capable and incapable of excluding the dye, respectively (Figure 1A:b). Moreover, we quantitatively evaluated the decline of living cell density in the cells in PSI. The percentage of living cells differed significantly between the cells in the vehicle (100%) and PSI (85%), P < 0.05 (Figure 1, A: c).

### Evaluation of a procedure of cellular fractionation

Using the hematoxylin-eosin (H&E) histological staining, we detected the eosinophilic PSI-induced inclusions in the cells with exposure to PSI for 48 h. Compared to the cells in the vehicle which were shown without no morphological change in eosinophilic structure (Figure 1B:a), the cells in PSI were shown with distinct eosinophilic inclusions (Figure 1B:b). Further, similar to inducible inclusions from PC12 cells in PSI for 24 h or MES cells in mitochondrial respiratory chain complex I inhibitor, rotenone for 72 h [19,24], the PSI-induced inclusions appeared with a focal, homogeneous shape and displayed two types of morphology: nucleus-binding and nucleus-free inclusions. It was assumed that some of the PSI-induced inclusions were observed in the cytoplasm of the remaining cells, whereas the others were extruded into the extracellular space after destruction of the host cells [25].

At the first stage of isolation, using the H&E histological staining, we detected the eosinophilic PSI-induced inclusions, collected in the fraction of initial pellets. By homogenization of the cells with 0.5% NP-40 at 37°C for 30 min and subsequent centrifugation at 80 × g for 15 min, an abundance of intact PSI-induced inclusions (Figure 1B:c) was yielded. In practice, we also increased the number of nucleus-free PSI-induced inclusions. There was a significant difference in the percentage of the nucleus-free to total PSI-induced inclusions between the fraction of cells in PSI (51%; Figure 1B:d, Column 1) and the fraction of initial pellets (69%; Figure 1B:d,Column 2), *P < 0.05.

At the second stage of isolation, using the H&E histological staining, we first detected the PSI-induced inclusions, collected in the temporary and final fractions of resulting pellets. By four time-dependent incubations of the initial pellets with 200 U/ml DNase I at 37°C for different lengths of time during the 24 h period, the increasing number of nucleus-free PSI-induced inclusions were easily yielded at the cost of the gradual decline of nucleus-binding PSI-induced inclusions, and of which, when the one of the time-dependent incubations ran out for the entire 24 h, the increase of nucleus-free PSI-induced inclusions instead of the decline of nucleus-binding PSI-induced inclusions could be most easily yielded. In detail, after the four time-dependent incubations for 1 h (Figure 1B:e), 6 h (Figure 1B:f), 12 h (Figure 1B:g) and 24 h (Figure 1B:h), the inclusive regions of nucleus (close arrow heads) but not the inclusive regions of inclusion (open arrows) within the nucleus-binding PSI-inclusions were progressively reduced. The percentage of the nucleus-free to total PSI-induced inclusions in the temporary fraction of resulting pellets which was collected after the one of the four time-dependent incubations of the initial pellets for 1 h (79%; Figure 1B:j Column 3) differed significantly from each of the percentages in the temporary fractions of resulting pellets which were collected after the other three of the four time-dependent incubations of the initial pellets for 6 h (80%; Figure 1B:j Column 4), 12 h (81%; Figure 1B:j Column 5) and 24 h (93%; Figure 1B:j Column 6), *P < 0.05. Interestingly, by the incubation of the initial pellets with 200 U/ml DNase I and 250 μg/ml RNase A for 24 h, a large quantity of nucleus-free PSI-induced inclusions instead of few nucleus-binding PSI-induced inclusions was observed in the final fraction of resulting pellets (Figure 1B:i). All percentages in the first four temporary fractions of resulting pellets (Figure 1B:j, Column 3-6) differed significantly from the percentage in the final one (97%; Figure 1B:j Column 7), **P < 0.05.

At the second stage of isolation, we detected the alpha synuclein (alpha-SYN) positive, eosinophilic inclusions, collected in the isolated fraction of PSI-induced inclusions, using the alpha-SYN immunostaining and the H&E histological staining, and then detected the relatively small amount of histone H1 present in the same fraction using the West blotting for histone H1, one of the most abundant proteins in the nucleus [26,27]. In general, subcellular particles, such as cellular debris and technical artifacts, were created after the homogenization at the first stage of isolation, and in particular, nuclear proteins were remained after the nucleus degradation at the second stage of isolation. Thus, by centrifuging the final fraction of resulting pellets in discontinuous Percoll-mediated 12%-35% gradients at 35,000 × g for 30 min, the intact PSI-induced inclusions could be easily divided from these subcellular particles and collected as preparation of the target fraction. On one hand, whether the PSI-induced inclusions were formed in the cells in PSI (Figure 1C:a and 1C:a') or isolated through cellular fractionation (Figure 1C:b and 1C:b'), they were stained as alpha-SYN positive, eosinophilic structures. On the other hand, in comparison to the intensive signal of blotted histone H1 in gel image and its high level in the column diagram, both of which were characteristic of the large amount of nuclear proteins for the samples of the cells in the vehicle or in PSI, the faint signal of blotted histone H1 in gel image and its low level in column diagram were characteristic of the trace of nuclear proteins for the sample of the isolated fraction of PSI-induced inclusions (Figure 1D:a and 1D:b). In the end, the pure intact PSI-induced inclusions were found in the isolated fraction.

### Two-dimensional (2-D) gel-based protein resolution

**Figure 2:**
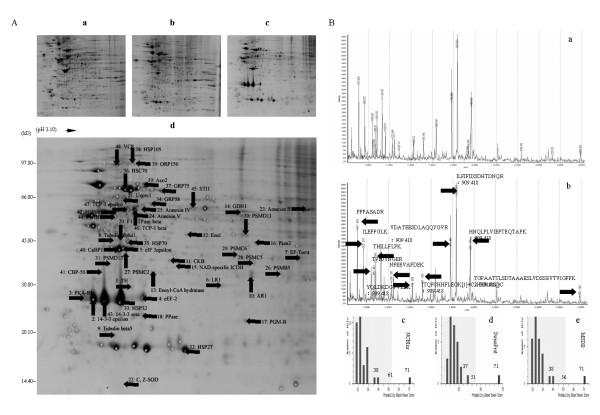
**Coomassie blue staining of proteins resolved in 2-D gel and identification of P4HB via PMF**. A, representative patterns of protein spots: a, cells in vehicle; b, cells in PSI; c or d, the isolated fraction of PSI-induced inclusions before and after protein gel spot picking from 2-D gel. B, identification of P4HB via PMF: a, an expanded full scan of PMF; b, the number of theoretically matched peptides and their sequences after preliminary identification via PMF against the single NCBInr database; c-e, comparable values of Mowse score after consistent identification via PMF against the multiple NCBInr, SwissProt, MSDB databases. Mowse score is -10×Log (P), where P is the probability that the observed match is a random event. Significance levels in the multiple databases are 61 in NCBInr branch, 51 in SwissProt branch and 56 in MSDB branch, respectively (p < 0.05). Close arrows indicate locations of the protein spots of interest in 2-D gel for identification, and theoretical sequences of the matched peptides for P4HB after identification.

We detected the protein spot pattern of the isolated fraction of PSI-induced inclusions using 2-D gel electrophoresis. Since aggresomes in cell culture models of PD act as disposal grounds for insoluble protein aggregates [4,11], we assessed proteome organization of the isolated fraction of PSI-induced inclusions by systematic analysis of protein complexes. Lysis buffer was added to the isolated fraction of PSI-induced inclusions to split inclusions, and an optimal formulation of rehydration solution was used to dissolve proteins from the split-products [28,29]. In conventional ultrasonic extraction, the enhancement of extraction efficiency is attributed to acoustic cavitation [20,30]. Thus, soluble proteins dissolved from the isolated fraction of PSI-induced inclusions were successfully resolved in 2-D gel as many individual protein spots. As with the two samples of proteins extracted from the homogenates of the cells in the vehicle and in PSI (Figure 2A:a and 2A:b), the sample of proteins extracted from the isolated fraction of PSI-induced inclusions was resolved on the left and central regions on 2-D gel (Figure 2A:c). That is the proteins displayed here are acidic and neutral proteins, as consistent with a previous observation that nervous system proteins in rat are mainly composed of acidic and neutral proteins [31]. In addition, most of the resolved proteins were within the molecular weight range of protein markers or the pH scale of isoelectric points (pI) or both of them. Undoubtedly, the proteomic profiling of the soluble proteins extracted from the isolated fraction was represented by the specific protein spot pattern in 2-D gel. Of the protein spots that were better focused in at least three of the four gels, one hundred and fourteen spots were reproducibly observed. Each spot was found not to differ significantly in terms of appearance, disappearance and shift. These protein spots of interest were selected for identification via peptide mass fingerprinting (PMF) (Figure 2A:d).

It is pointed out that values of both molecular mass and pI supplied in a matrix-assisted laser desorption/ionization time-of-flight (MALDI-TOF) mass spectrometry (MS) data file are usually theoretical. Some of the spots identified as proteins were found to alter their positions across 2-D gel in the present experimental condition. In general, the observed difference most probably reflects mechanisms for the augmentation of ubiquitin fusion-protein expression in eukaryotes when proteasomes are inhibited beyond a threshold level [32-34].

### PMF-base protein identification

We detected experimental PMF spectra of the target protein spots using MALDI-TOF MS, and then PMF data for protein identification using two on-line proteomic search engines. Preliminarily, a total of fifty six proteins were identified after submission of their PMFs to the ProPound search engine against the single NCBInr database (shown in Additional file 1). Furthermore, forty eight of the fifty six proteins were consistently identified after submission of their PMFs to the Mowse score-based Mascot search engine against the multiple NCBInr, SwissProt, MSDB databases (shown in Additional file 1). For example, prolyl-4-hydroxylase beta subunit (P4HB) identified via its PFM against the single database is shown with a group of MS data, such as 32.4% sequence coverage, 0.000 expectation, 8 matched peptides and theoretic sequences of the matched peptides (Figure 2B:a and 2B:b). After consistent identification via its PFM against the multiple databases, it was shown with its three comparable score values, viz., a top score value of 71 (> 61) compared to a runner up value of 38 (< 61) for NCBInr branch, 71 (> 51) compared to 37 (< 51) for SwissProt branch, and 71 (> 56) compared to 38 (< 56) for MSDB branch (Figure 2B:c to 2B:e). The protein was ultimately determined.

**Figure 3:**
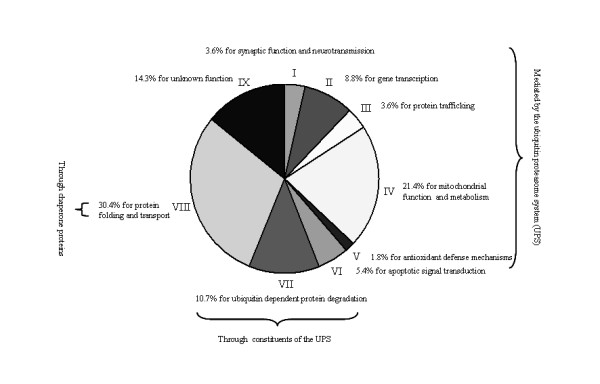
**Functional categories of proteins identified in PSI-induced inclusions**. This pie chart shows that all the identified proteins are classified into at least nine accepted categories as follows: 3.6% for synaptic function and neurotransmission (I); 8.8% for gene transcription (II); 3.6% for protein trafficking (III); 21.4% for mitochondrial function and metabolism (IV); 1.8% for antioxidant defense mechanisms (V); 5.4% for apoptotic signal transduction (VI); 10.7% for ubiquitin dependent protein degradation (VII); 30.4% for protein folding and transport (VIII); and 14.3% for unknown function (IX). When a protein could be divided into more than one class of functions, it was indicated as the best known class.

Among the fifty six proteins, twenty were found to be previously reported components of LBs. These components of LBs identified in the isolated fraction of PSI-induced inclusions are listed as follows: two key proteins in the synthesis of neural transmitter dopamine (DA), i.e. tyrosine hydroxylase (TH) and 14-3-3epsilon (No.1-2) [35,36]; two microtubulins, i.e. tubulin alpha1 and tubulin beta5 (tubb5) (No.8-9) [37]; two mitochondria matrix proteins, i.e. ATP synthase beta subunit (F1-ATPase beta) and ubiquinol-cytochrome c reductase core protein 1 (Uqcrc1) (No.20-21) [38,39]; Cu, Zn superoxide dismutase (C, Z-SOD; No.22) [40,41]; six proteasomal subunits, i.e. proteasome subunit beta 5, proteasome 26 S subunit ATPase 2 (PSMC2), proteasome 26 S subunit ATPase 5, proteasome 26 S subunit ATPase 6, proteasome 26 S subunit non-ATPase 11 and proteasome 26 S subunit non-ATPase 13 (No.26-31) [23,42]; and seven chaperone proteins, i.e. 27-kD heat shock protein 1 (HSP27; No.32) [43], heat shock protein 32 (HSP32; No.33) [44], 70-kD heat shock protein 1A/1B (HSP70; No.35) [23], 71-kD heat shock cognate protein (HSC70; No.36) [11], 14-3-3zeta (No. 43) [45,46], T-complex polypeptide 1 beta subunit (TCP-1beta; No.46) [47] and Valosin-containing protein (No.48) [48]. Comparatively, the twenty eight proteins were newly identified and, if selected for further study, may provide novel clues to our understanding of the potential proteomic candidates of aggresomes in response to proteasome inhibition by PSI. For the other eight proteins which were incompletely determined by the Profound search engine using the single NCBInr database alone (shown in Additional file 2), maybe it's preferable for them to be identified in the future since update on construction of a protein reference database has been considered as a discovery resource for proteomics [19-21]. These proteins, most of which were recognized as a profile of proteins within cellular processes mediated by the UPS, a profile of constituents of the UPS and a profile of chaperone proteins, are classed into at least nine accepted categories [4,19,20]. Functional classification of the fifty six proteins and their percentages are shown in Figure 3. Given that in eukaryotes, aggresomes sequester aberrant and potentially cytotoxic proteins from the a wide range of cellular processes mediated through the UPS and also serve as sites for the recruitment and concentration of constituents of the UPS and chaperone proteins [11], to date the protein dataset are at the level of proteomics valuable for characterization of aggresomes, induced in PC12 cells by PSI and also in vitro by many other proteasome inhibitors such as lactacystin or MG132 [4,14]. Moreover, the profile of chaperone proteins, accounting for the predominant portion of the protein dataset and classed into the category of protein folding and transport, have been found in available literatures to share some similarity between protein families and specialize in various cellular localization resources (summarized in Additional file 3). They are at the level of bioinformatics valuable for providing cellular information on relationship between chaperone proteins, known as aggresome related proteins, and aggresomes, known as intermediate compartments [11].

### Validation

**Figure 4:**
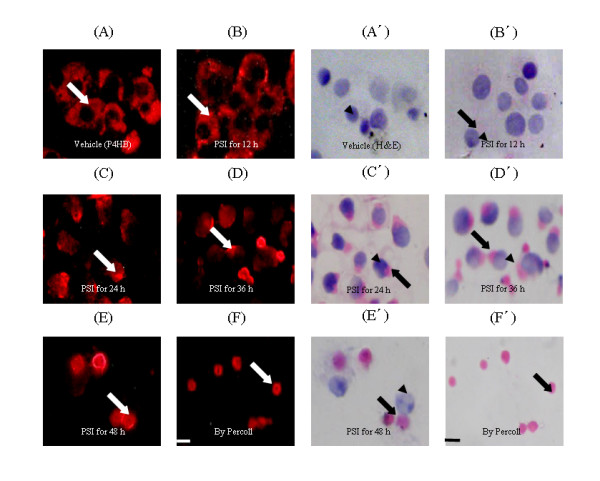
**P4HB immunostaining for and H&E histological staining of PSI-induced inclusions developed in PC12 cells after proteasome inhibition by PSI**. A and A', cells in the vehicle; B-E and B'-E', cells in PSI for 12, 24, 36 and 48 h; F and F', a fraction of inclusions isolated from the cells in PSI for 48 h. Development of inclusions in the cells at the end of indicated points of PSI exposure is indicated as closed arrows, and nuclei and juxta-inclusion nuclei indicated as closed arrow heads. Scale bar, 10 μm.

Proteins identified via PMF need validation to show their biological presence. A candidate selected for validation was determined by three criteria as follows: it was significantly focused as an individual spot in 2-D gel and detected as with a desirable group of MS identification data; it has not been reported in published literature on LBs; it matches to commercial specific antibody. Thus, we sought to determine whether P4HB, a member of the protein disulfide isomerase (PDI) family in endoplasmic reticulum (ER) lumen, was present in PSI-induced inclusions in PC12 cells with proteasome inhibition, and if so, whether it redistributed from its cellular location resource during the induction of inclusions. We validated P4HB in the developmental process of PSI-induced inclusions in PC12 cells in PSI using the P4HB immunostaining and the H&E histological staining. Compared to the uniform immunostaining throughout cytoplasm of the cells in the vehicle (Figure 4A), P4HB tended to converge to juxtanuclear regions in the cells in PSI (Figure 4B to 4E). Specifically, after proteasome inhibition by PSI for 12 h, P4HB was present with immunostaining of asymmetric structures throughout cytoplasm and around nuclei (Figure 4B); after the proteasome inhibition for 24 h or 36 h, P4HB was present with immunostaining of granular and elliptical perinuclear structures (Figure 4C and 4D); after the proteasome inhibition for 48 h, P4HB was present with immunostaining of spherical structures (Figure 4E), which was also shown in the isolated fraction of PSI-induced inclusions (Figure 4F). Further, compared to hematoxylin staining of ovoid nuclei of the cells in the vehicle (Figure 4A'), P4HB-captured structures stained readily with eosin were present in the cells in PSI for the different lengths of time (Figure 4B' to 4E') and in the fraction of PSI-induced inclusions isolated from the cells in PSI for 48 h (Figure 4F').

## Discussion

In this study, proteasome inhibition by PSI induced cell death and formation of inclusions in PC12 cells. An end point cell death estimated using trypan blue exclusion assay confirmed the effect of proteasome inhibition by PSI on survival of the cells. Cytoplasmic eosinophilic PSI-induced inclusions in the cells were immunostained to visualize alpha-SYN positive structures. Experimental observations have been providing evidence that application of proteasome inhibitors such as lactacystin and (or) PSI to PC12 cells is closely associated with both cell death and formation of inclusions [49-52]. Nevertheless, low doses of PSI which produces the highest number of inclusions in PC12 are not enough to induce massive cell death in the cells, whereas increasing its doses lead to mass damage of the cells which does not allow the visualization of inclusions [24,52], as shown similarly in PC12 cells with exposure to methamphetamine [9]. Thus, the administration of PSI to PC12 cells we selected could induce the abundance of cytoplasmic inclusions as well as a massive amount of cell death. Besides, morphological maturation of the PSI-induced inclusions could allow us to appreciate how their isolation was achieved.

In the study, we developed a three-process fractionation procedure to isolate a fraction of PSI-induced inclusions from the cell culture model of PD, and thereafter characterized a proteomic feature of the isolated fraction. Traditional fractionation for isolating any population of organelles from cell cultures consists of two major stages: homogenization of cell cultures to release various populations of intact organelles, and fractionation of homogenate to isolate the population of targeted organelles [22]. At the first stage of isolating PSI-induced inclusions, a first process of fractionation procedure was used to separate the abundance of nucleus-binding and the nucleus-free PSI-induced inclusions into the fraction of initial pellets. At the second stage of isolation, a second process of fractionation procedure was used to increase the nucleus-free PSI-induced inclusions at the cost of declining the nucleus-binding PSI-induced inclusions, after which the whole PSI-induced inclusions were enriched into the fraction of resulting pellets; in the next isolation, a third process of fractionation procedure was used to purify PSI-induced inclusions into the preparation of target fraction. In the case of the target fraction that should be prepared as starting material sufficient for proteomic analysis, the 2-D gel-based protein resolution and the PMF-based protein identification were used to define a proteomic feature of the isolated fraction of PSI-induced inclusions. Of a total of fifty six proteins identified, twenty proteins such as TH were found to be previously reported protein components of LBs, twenty eight proteins such as eukaryotic elongation factor 2 (eEF-2) were newly identified, and the other eight proteins were in lower confidence for information about protein designation. These proteins, most of which were recognized as a profile of proteins within cellular processes mediated by the UPS, such as tubulin alpha 1, a profile of constituents of the UPS, such as PSMC2, and a profile of chaperone proteins, such as HSP27, are classed into at least nine accepted categories. In addition, P4HB, an ER member of the PDI family, was biologically validated in the developmental process of PSI-induced inclusions in PC12 cells after proteasome inhibition by PSI.

In nerve endings and particularly brain neurons, it is likely that proteins are easily damaged owing to limitation of intrinsic ability for repair and (or) regeneration, level of relatively high oxygen and elevated metabolic rate, and enzymatic- and auto-oxidation of neurotransmitters such as DA. Protein degradation via the UPS exerts an effect on the removal of damaged proteins that are extensively linked to cellular processes, such as synaptic function and neurotransmission (I), gene transcription (II), protein trafficking (III), mitochondrial function and metabolism (IV), antioxidant defense mechanisms (V), apoptotic signal transduction (VI) [4]. Cooperation of constituents of the UPS (VII) with chaperone proteins (VIII) in protein degradation plays the central role in these processes [53,54].

I. TH identified in the study is regulated at two levels: a long-term regulation involves changes in enzyme synthesis for several days, while a short-term regulation of enzyme activity is arrested by direct phosphorylation of the protein within several minutes [55]. Alpha-SYN diminishes TH phosphorylation and activity in stably transfected dopaminergic cells [56]. Given that alpha-SYN aggregation is induced by proteasome inhibitors [24] and that TH is degraded through the UPS [57,58], it is not coincidence that TH colocalizes with alpha-SYN in LBs [35].

II. eEF-2 identified in the study is one of the gene transcription factors whose cellular level in protein turnover process is dependent on the UPS [59]. Proteasome inhibition by proteasome inhibitors in cells activates up-regulation of specific members of transcription factor families controlling cellular stress response [60]. Transcription factors may be trapped by cytoplasmic protein aggregates in response to proteasome inhibition [61,62]. In addition, eEF-2 is phosphorylated by specific eEF-2 kinase causing its complete inactivation [63], and relevance of transient eEF-2 kinase to the UPS may define cellular levels of eEF-2 between dephosphorylation and phosphorylation [64]. Potentially phosphorylated eEF-2 might acquire aggregation competence in PC12 cells after proteasome inhibition by PSI.

III. Tubulin alpha1 and tubb5 identified in the study not only colocalize with alpha-SYN in LBs or other alpha-SYN positive pathological structures, but also initiate alpha-SYN fibril formation under pathological conditions in vitro [37]. The binding between parkin and tubulin led to increased ubiquitination and accelerated degradation of alpha- and beta-tubulins in vitro [65].

IV. The UPS can influence mitochondria under pathologic conditions [66]. Proteasome inhibition by proteasome inhibitors not only increases the generation of reactive oxygen species (ROS) in mitochondria [67,68], but also activates the compartmentalization of mitochondrial proteins in aggresomes [69]. The two mitochondrial proteins identified in the study and found as components of LBs, F1-ATPase beta and Uqcrc1, might be damaged by mitochondrial ROS.

V. Antioxidant enzyme C, Z-SOD identified in the study is the most notable component of antioxidant protective defense system in response to oxidative stress by both SOD mechanism and hydrogen peroxide system mediated-protein modification [70-72]. Proteasome inhibition by proteasome inhibitors has been shown to generate oxidative stress in vitro and potentiate compensatory up-regulation of antioxidants as a protective mechanism against oxidative stress [14,73,74]. The oxidative modification of C, Z-SOD is associated with PD [75].

VI. Apoptosis is the major mechanism of neuronal death in PD. Apoptotic response is quantified by all members of the annexin family, especially annexin V [14,76]. Three members of the annexin family were identified in the study. Proteasome inhibition by proteasome inhibitor MG-132 in a dopaminergic neuronal cell model (N27 cells) rapidly depolarized mitochondria independent of ROS generation to activate the apoptotic cascade [77]. Aggresomes may be viewed as the wrong cellular compartments where apoptotic cascade proteins are sequestered after detrimental effects of proteasome inhibition by proteasome inhibitors on mitochondrial homeostasis [78].

**Figure 5:**
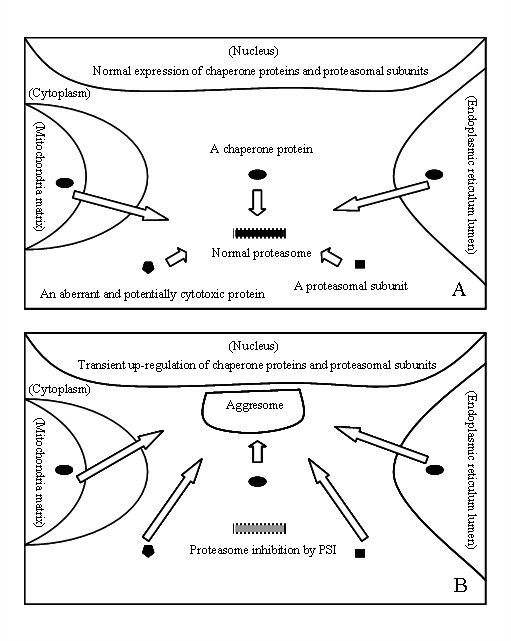
**Potential mechanisms by which constituents of the UPS and chaperone proteins may affect development of PSI-induced inclusions in PC12 cells after proteasome inhibition by PSI**. A, relevance of chaperone proteins in cytoplasm, ER and mitochondria resources to cytoplasmic constituents of the UPS establishes multiple routes of the UPS to remove aberrant and potentially cytotoxic proteins [2,87]. The interaction between constituents of the UPS and cytoplasmic chaperon proteins might present one route to protein degradation in cytoplasm, UPP by which its target proteins serving as protein substrates of enzyme in the cytoplasm are directed by chaperone proteins to proteasomes [2]. The coordination between constituents of the UPS and ER chaperone proteins might present another route to protein degradation in ER, ERAD by which its target proteins serving as protein substrates of enzyme in lumen of the ER are bound to chaperone proteins, retro-translocated through a multi-protein translocon complex across ER membrane, get ubiquitinated by ubiquitin ligases, and are subsequently targeted for degradation by proteasomes [87]. New data support the notion that a route of the UPS similar to that observed in ERAD occurs in mitochondria and therefore the name of MAD has been proposed [87]. B, constituents of the UPS in cytoplasm and chaperone proteins in cytoplasm, ER and mitochondria resources, together with the protein substrates of the UPS, could be recruited to PSI-induced inclusions formed in PC12 cells after proteasome inhibition by PSI.

VII. Proteasomal subunits are in progress of the tightly coordinated expression and integrated assembly under physiological conditions [79,80]. However, under proteolytic stress conditions elicited by proteasome inhibitors, proteasomal subunits display the alterable profile of expression and composition [14,81]. For example, treatment of cells with proteasome inhibitors activates a transient up-regulation of mRNAs of all proteasomal subunits [82]. The alteration might represent a regulatory feedback mechanism attempting to reverse proteasome inhibition [80,83], but if the compensatory regulation fails to establish affection, proteasomal subunits are recruited to aggresomes to ubiquitinate and degrade protein (Figure 5) [11,42]. One beta subunit of 20 S proteasome core particle and five ATPase-dependent and ATPase-independent subunits of 19 S proteasome regulatory particle were identified in the study, but it is not known whether proteasomal subunits rich in LBs exist as individual subunits or as part of the intact complex [23,84,85].

VIII. Cells usually express diverse family members of chaperones and different versions of a family member of chaperones to block protein aggregation [2,86]. The chaperone proteins identified in the study own their specialized protein similarities. The intra- and inter-specific species of chaperones are listed by chaperone proteins rich in LBs, such as members of the HSP70 family (in particular, HSP70 and HSC70) and members of the small HSP family (in particular, HSP27 and HSP32) [11,23,43,44]. In substance, proteasome inhibition by proteasome inhibitors increased initial and concerted expression of major types of chaperone proteins in cytoplasm, ER and mitochondria [61,87,88]. The compensatory induction of chaperone proteins can be beneficial since aggresomes are supposed to recruit both constituents of the UPS and chaperone proteins to facilitate protein clearance within the structures [11].

A function for chaperone proteins in targeting aberrant and potentially cytotoxic proteins for degradation via the UPS has been established in various ways [89]. In substance, act of the UPS might depend on the cellular compartment-localized chaperone proteins to guide it along proper pathways [89,90]. What this means is that chaperon proteins in cytoplasm (in particular, TCP-1beta) are required for the ubiquitin proteasome pathway (UPP) [91], chaperone proteins in ER (in particular, 150-kD oxygen regulated protein) required for the ER associated degradation pathway (ERAD) [92], and chaperone proteins in mitochondria (in particular, HSP60) required for the mitochondria-associated degradation pathway (MAD) [87]. Diversity of chaperones among cellular localization resources is listed by spatially restricted subset of chaperone proteins, such as cytoplasmic HSP27, ER luminal 78-kD glucose-regulated protein (GRP78) and mitochondrial HSP60 [69,93-95]. Relevance of chaperone proteins in cytoplasm, ER and mitochondria to cytoplasmic constituents of the UPS establishes multiple routes of the UPS to remove aberrant and potentially cytotoxic proteins [2,87], but in response to proteasome inhibition, constituents of the UPS in cytoplasm and chaperone proteins in cytoplasm, ER and mitochondria resources, together with the protein substrates of the UPS, are induced and recruited to aggresomes (Figure 5) [11,87,90].

P4HB, also known as PDI [96], is a highly unusual multifunctional polypeptide [97]. As with calcium-binding proteins and GRPs which are two members of the PDI family [92,98], P4HB acts through ERAD to eliminate proteins unable to adopt native structure after translocation into the ER [99]. On the basis of biological information retrieved from the studies with two model animals, Pongo abelii (accession No., **gi|62287145|sp|Q5R5B6.1**) and Macaca fuscata fuscata (accession No., **gi|110815912|sp|Q2HWU2.1)**, P4HB has been defined as follows: it acts as a structural subunit of various enzymes, such as proly-4-hydroxylase; it catalyzes the formation, breakage and rearrangement of disulfide bonds; it serves as a natural protector against protein aggregation at high concentration and for protein self-assembly and aggregation at low concentration. Given that up-regulation of the PDI family in dopaminergic neurons might contribute to formation of LBs [100], and that the formation of LBs may act to delay the initiation of neuronal death and slow progression in sporadic PD, the members of the PDI family identified in the study might expand the defensive role for chaperone proteins in the development of PSI-induced inclusions.

Aggresomes are often enriched with aggresome-related proteins such as chaperone proteins [11]. Next, our task is to establish an immunoblot method for semi-quantitative or quantitative screening of the ten chaperone proteins which were identified in the study but have not been reported in available literatures on LBs. Also, further studies will be required to demonstrate whether or not these proteomic candidates are recruited into aggresomes in response to proteasome inhibition by many other proteasome inhibitors (in particular, lactacystin or MG132). The importance of validation experiments in proteomic analysis and the collection of comparable data from further studies will motivate us to reveal aggresomes in protein content.

## Conclusions

Taken together, the fifty six proteins identified by proteomic analysis are associated with the isolated fraction of PSI-induced inclusions in PC12 cells after proteasome inhibition by PSI, and at this stage of the study, it is speculated that proteomic characterization of an isolated fraction of PSI-induced inclusions in PC12 cells might offer clues to appearance of aggresomes serving as a cellular defensive response against proteasome inhibition.

## Methods

### Chemicals

Unless specified otherwise, analytical reagents and reagents of cell culture grade were purchased from Amersham Biosciences (Uppsala, Sweden). PSI, N-benzyloxycarbonyl-Ile-Glu (O-t-butyl)-Ala-Leu-al, was from EMD Biosciences (an affiliate of Merck Chemicals, Darmstadt, Germany). Cell culture plastics, culture media and supplying chemicals were from Gibco (Grand Island, NY, USA). DNase I and RNase A were from TaKaRa Biotechnology (Dalian, China). Percoll (density 1.131 g/ml) and proteinase inhibitors [4-(2-aminoethyl) benzenesulfonyl fluoride, pepstatin A, E-64, bestatin, leupeptin, and aprotinin] were from Sigma (St. Louis, USA). Mouse anti-rat alpha-SYN monoclonal antibody (4D6: sc-65500) and mouse anti-rat histone H1 monoclonal antibody (sc-56694) were from Santa Cruz Biotechnology (California, USA); mouse anti-rat P4HB monoclonal antibody (MAB2073) was from Chemicon (Fuji, Japan). Cy3-conjugated goat anti-mouse secondary antibody (00009-1) was from Proteintech Group (Chicago, USA).

### Cell culture

PC12 cells were purchased from Cell Bank of the Chinese Academy of Science (Shanghai, China), and their culture was carried out as described elsewhere [25].

### Proteasome inhibition and cell survival

PC12 cells, seeded at a density of 1 × 10^5 ^cells/ml in 96-well plates and incubated for 24 h, were exposed to 10 μM PSI or equal volume of its vehicle, 0.1% DMSO (V/V) in culture medium, for 48 h [25]. Then, they were centrifuged at 1000 rpm (or 93 × g), 4°C for 5 min [101] and stained with 50 μl of 1% trypan blue (W/V) for 2 min [102]. After supernatant was removed from the cell cultures, living cells at a density of at least 100 cells per field were viewed under an upright fluorescence microscope with IF550 green interference filter in the light path. The number of living cells was counted in nine random fields (three fields per well of a 96-well plate, three different wells) and expressed as a percentage [103,104]. Chi square test (χ^2 ^test) was used to determine whether a percent difference was significant between the cells without and with PSI exposure. Probability value (P) < 0.05 was accepted as significant. Measurements were repeated at least 3 times.

### Formation and isolation of PSI-induced inclusions

Formation of PSI-induced inclusions and their isolation through cellular fractionation were carried out as described elsewhere [25]. Cells exposed to 10 μM PSI were subjected to the H&E histological staining to monitor appearance of the PSI-induced inclusions. Progress in the isolation of PSI-induced inclusions included two sequential stages: separation for an abundant fraction of intact PSI-induced inclusions, and purification for preparation of the target fraction. At the first stage of isolation, briefly, cells in PSI for 48 h were scraped, collected and washed in ice-cold Tris-buffered saline solution (TBS; pH 7.4) and ice-cold 8% sucrose (W/V) in 0.1 × TBS one after the other. The fraction of the cells was frozen repeatedly with liquid nitrogen, and homogenized with a certain amount of lysing buffer consisting of 1 mM Hepes (pH 7.2), 0.5 mM MgCl_2_, 0.5% NP-40 (V/V), 0.1% beta mercaptoethanol (V/V), and 1% proteinase inhibitors (V/V). After repeated suspension and vibration, the lysate was incubated at 37°C for 30 min until the cells were thoroughly homogenized. Initial pellets were collected by low speed centrifugation at 80 × g for 15 min, washed in ice-cold buffer solution L consisting of 1 mM Hepes, 0.5 mM MgCl_2 _and proteinase inhibitors, and recollected by centrifugation at 836 × g.

At the second stage of isolation, the fraction of initial pellets was subjected to the H&E histological staining to monitor presence of the eosinophilic PSI-induced inclusion. In the next isolation, four equal aliquots removed from the fraction of initial pellets were incubated in 200 U/ml DNase I alone in DNase I buffer plus proteinase inhibitors at 37°C for 1 h, 6 h, 12 h and 24 h, respectively, whereas another equal aliquot from the fraction was incubated in the DNase I solution with addition of 250 μg/ml RNase A at 37°C for 24 h. In their respective incubations, initial pellets were repeatedly suspended and vibrated vigorously. After incubation, the first four temporary fractions of resulting pellets and the final one were collected by centrifugation at 836 × g, washed with 0.32 M sucrose in TBS, and subjected to the H&E histological staining to monitor presence of the eosinophilic PSI-induced inclusion.

The final fraction of the resulting pellets was diluted to 600 μl using 12% Percoll in TBS (V/V) and overlaid on equal volume of 35% Percoll in TBS. The material band just below the interface of the sample and 35% Percoll was removed, collected by centrifugation at 35,000 × g, washed in 250 mM sucrose in 10 mM Tris (pH 8.0), and recollected by centrifugation at 4000 × g. This isolated fraction of PSI-induced inclusions was the preparation of target fraction. The target fraction was subjected to the α-SYN immunostaining and the H&E histological staining to monitor presence of the PSI-induced inclusion, and then subjected to the Western blotting for histone H1 to monitor contamination of nuclear protein content.

### Assessment of PSI-induced inclusions

Eosinophilic PSI-induced inclusions were examined by an experienced observer not familiar with the sample identity. The cells exposed either to the vehicle or to PSI, the fraction of initial pellets, and the five fractions of resulting pellets were all subjected to the H&E histological staining, as previously described [25]. The number of PSI-induced inclusions was counted in nine random fields (three fields per section, three different sections on slide), and expressed as a percentage of nucleus-free to total PSI-induced inclusions. The χ^2 ^test was used to determine whether there was significant difference in the percentage between every two objects of comparison as follows: between the cells in PSI and the fraction of initial pellets, between the first one temporary fraction of resulting pellets and each of the other three temporary fractions of resulting pellets, and between each of the four temporary fractions of resulting pellets and the final fraction of resulting pellets. P < 0.05 was accepted as significant. Measurements were repeated at least three times.

The isolated fraction of PSI-induced inclusions, together with a control (cells in PSI), was first subjected to the alpha-SYN immunostaining and then the H&E histological staining. In the application of immunostaining for alpha-SYN, briefly, the isolated fraction of PSI-induced inclusions was spun on slides and fixed in 4% paraformaldehyde (W/V). Sections on the slides were permeabilized with phosphate-buffered saline containing Triton X-100 (PBS-T; V/V), blocked in 5% bovine serum albumin (BSA; V/V) in PBS for 1 h, incubated with primary antibody at 1:50 dilution in PBS at 37°C for 3 h and incubated with secondary antibody at dilution 1:100 in PBS-T at 37°C for 3 h, and mounted with 80% glycerol (V/V). The dilutions of primary and secondary antibodies were according to the reference to manufacturer's instructions and the description in literature [105]. Control sections were examined, as described above, excluding primary antibody in PBS. Measurements were repeated at least three times.

The isolated fraction of PSI-induced inclusion, together with two controls (cells in PSI and in the vehicle), was subjected to the Western blotting for histone H1. Briefly, the isolated fraction of PSI-induced inclusion was frozen repeatedly with liquid nitrogen, and dissolved with a certain amount of lysing solution consisting of 30 mM Tris, 7 M urea, 2 M thiourea, 4% 3-[(3-cholamidopropyl) -dimethylammonio]-1-propanesulphonate (CHAPS; W/V), 60 mM dithiothreitol (DTT), and proteinase inhibitors. The mixture was incubated, concussed, and collected by centrifugation at 25,000 × g, 4°C for 30 min. Approximately 20 μg of protein extracts adjusted to a total volume of 20 μl was resolved by polyacrylamide gel electrophoresis in a gel buffer. A 0.5-mm-thick discontinuous polyacrylamide gel system without stacking gel layer was determined by 0.1% sodium dodecyl sulfate (SDS; W/V), 15% total gel (T; W/V), and 2.6% cross-linking agent (C; W/W). Protein markers with a range of 23-132 kD were used to measure molecular masses of the resolved proteins. After the gel was transferred to a polyvinylidene difluoride membrane, the membrane was blocked in 5% BSA in PBS-T at 37°C for 1 h, incubated with primary antibody at 1:100 dilution in 1% BSA in PBS-T at 4°C overnight and incubated with secondary antibody at 1:500 dilution in 1% BSA in PBS-T at 37°C for 2 h. The blotted membrane was scanned using a Typhoon 9400 fluorescent laser scanner (Emission filter at 580 BP Cy3 mode, wavelength at 532 nm; Amersham Biosciences) with Typhoon Scanner Control Version 3.0 (Amersham Biosciences). The digitalized image of the immunoblot was analyzed with DeCyder Image QuantTM Version 5.0 (Amersham Biosciences). Bands of histone H1 in image were densitometrically quantified using ImageQuant Version 5.0 (Amersham Biosciences). Dilutions of both primary and secondary antibodies were according to the reference to manufacturer's instructions and the description in literature [26,27]. Control blotting membrane was carried out, as described above, excluding primary antibody in 1% BSA in PBS-T. Measurements were repeated at least three times.

### Protein extraction, 2-D gel electrophoresis and image analysis

Protein extraction, 2-D gel electrophoresis and image analysis were carried out as described elsewhere [25,106]. Briefly, the isolated fraction of PSI-induced inclusion, frozen repeatedly with liquid nitrogen, was dissolved with a lysis buffer consisting of 30 mM Tris, 7 M urea, 2 M thiourea, 4% CHAPS, 60 mM DTT, 2% pharmalyte (Ph 3.0-10.0; V/V) and proteinase inhibitors. The mixture was incubated, sonicated, and collected by centrifugation at 25,000 × g, 4°C for 30 min. A certain amount of protein extracts prepared from the isolated fraction of PSI-induced inclusion, together with two controls prepared from cells in PSI and the vehicle, were demineralized with 2-D Clean-Up kit, re-dissolved with a rehydration solution consisting of 8 M urea, 2% CHAPS (W/V), 0.6% DTT (W/V), and 0.5% immobilized pH gradients buffer (V/V), and applied to a non-linear Immobiline™ DryStrip IPG gel strip (pH 3.0-10.0; 24 cm; Amersham Biosciences). The first dimension electrophoresis was run on an Ettan IPGphor II Isoelectric Focusing Unit (50 μA per strip; Amersham Biosciences). The strip was then equilibrated in a loading solution consisting of 50 mM Tris-HCl (pH 8.8), 6 M urea, 2% SDS, 30% glycerol, 2% DTT and a trace of bromophenol blue, re-equilibrated in the same loading solution including 4% (W/V) iodoacetamide but excluding DTT, and placed onto the top of polyacrylamide gel (24 × 20 cm^2^). A 1-mm-thick continuous polyacrylamide gel system without stacking gel layer was determined by 0.1% SDS, 12.5% T, and 2.6% C. The protein markers were used to mark positions of proteins resolved in gel. The second dimension electrophoresis was run on an Ettan DALT six electrophoresis unit (2 W per gel, 600 V, 400 mA; Amersham Biosciences). Coomassie blue-stained gels were scanned using an Umax CE scanner (Amersham Biosciences) with Image Master Labscan version 3.01b (Amersham Biosciences). The digitalized image of the gels was analyzed with Image Master 2-D Evolution version 2003.02 (Amersham Biosciences). Spots in images were densitometrically quantified and statistically evaluated by pattern analysis of computer-assisted pit assessment to detect whether spots appeared reproducibly significant in at least 3 of the 4 gels. These protein spots of interest were selected for MS analysis. If a spot was not detected in at least 3 of the 4 gels, it was considered to be negligible in the present experimental condition.

### In-gel digestion, MS and database search

In-gel digestion, MS and database search were carried out as described elsewhere [25,107]. Briefly, using an Ettan Spot Picker robotic workstation (Amersham Biosciences), the protein spots of interest were automatically selected, excised and placed in 96-well plates at a gel plug per well. Using an Ettan TA Digester robotic workstation (Amersham Biosciences), the gel plugs were twice destained, dehydrated, desiccated and incubated in 10 μl of 1 μg/μl modified porcine trypsin at 4°C overnight, and after which the digested peptide fragments were extracted twice and the two combined extracts were desiccated in other 96-well plates. Using an Ettan Spotter robotic workstation (Amersham Biosciences), 0.3 μl of the digested peptide fragments and an equal volume of 4 μg/μl α-cyano-4-hydroxy-transcinnamic acid were deposited on the surface of a sample slide (Amersham Biosciences). The pre-mix was allowed at room temperature to facilitate uniform dispersion of the digested peptide fragments in a large mix.

Signals of MS were recorded in the positive ion reflection mode on a MALDI-TOF Pro workstation (Amersham Biosciences). The preset threshold values suitable for acquiring and processing MS were referred to our previous requirements [25]. Additionally, the most intensive signals of MS had to be characterized by at least 3 to 5 strongest peaks. Such an additional criterion can efficiently distinguish the target protein from the interference background, since experimental spectra for protein identification consist of the mass and intensity pairs, one pair for each peak [108].

Protein identification via PMF was initially accomplished using the ProFound search engine against the latest version of the single NCBInr database (fully integrated and included in the Pro workstation or at http://prowl.rockefeller.edu/prowl-cgi/profound.exe), and was further enhanced using Mowse score-based Mascot search engine against the latest version of the multiple NCBInr, SwissProt, MSDB databases http://www.matrixscience.com/search_form_select.html. In the initial identification, the requirements of evaluation standard were allowed as follows: sequence coverage of at least 20%, expectation as much as 0.05, a set of at least 5 matched peptides, and a gap of at least 3 peptides between the accepted protein candidate and the first excluded one in the list of protein candidates provided by the single NCBInr database [25]. For the enhancement of identification, except that mass tolerance at ± 0.2 D as a preset start-up value of searching parameter for the single database is different from mass tolerance at ± 0.1 D for the multiple databases, the others were identical in the two types of protein databases [25]. A protein, if assigned as the identical protein candidate against the two types of protein databases, was ultimately determined.

### Immunostaining

Cells exposed to PSI for 12, 24, 36 and 48 h, respectively, and a fraction of inclusions isolated from cells in PSI for 48 h were first subjected to the P4HB immunostaining and then the H&E histological staining. The dilutions of both primary (1:40) and secondary (1:100) antibodies were carried out according to the reference to manufacturer's instructions and the description in literature [109].

## Abbreviations

2-D: two-dimensional; alpha-SYN: alpha synuclein; eEF-2: eukaryotic elongation factor 2; χ^2 ^test: Chi square test; BSA: bovine serum albumin; C: cross-linking agent. CHAPS: 3-[(3-cholamidopropyl)-dimethylammonio]-1-propanesulphonate; C, Z-SOD: Cu, Zn superoxide dismutase; DA: dopamine; DMSO: dimethyl sulfoxide; DTT: dithiothreitol; ER: endoplasmic reticulum; ERAD: the ER associated degradation pathway; F1-ATPase beta: ATP synthase beta subunit; H&E: hematoxylin-eosin; HSC: heat shock cognate protein; HSP: heat shock protein; LBs: Lewy bodies; MAD: the mitochondria-associated degradation pathway; MALDI-TOF: matrix-assisted laser desorption/ionization time-of-flight; MS: mass spectrometry; P: probability value; P4HB: prolyl-4-hydroxylase beta subunit; PBS-T: phosphate-buffered saline containing Triton X-100; PD: Parkinson's disease; PDI: the protein disulfide isomerase; pI: isoelectric points; PMF: peptide mass fingerprinting; PSI: synthetic proteasome inhibitor; PSMC2: proteasome 26 S subunit ATPase 2; ROS: reactive oxygen species; SDS: sodium dodecyl sulfate; SNpc: the substantia nigra pars compacta; T: total gel; TBS: Tris-buffered saline solution; TCP-1beta: T-complex polypeptide 1 beta subunit; TH: tyrosine hydroxylase; tubb5: tubulin beta5; UPS: ubiquitin proteasome system; Uqcrc1: ubiquinol-cytochrome c reductase core protein 1; UPP: the ubiquitin proteasome pathway.

## Authors' contributions

XL developed a three-process fractionation procedure to isolate a fraction of PSI-induced inclusions, focused on MS analysis, and drafted the manuscript. YZ developed methods for 2-D gel-based protein resolution and PMF-based protein identification. PX performed the alpha-SYN immunostaining and the H&E histological staining. JP performed the West blotting for histone H1. YH performed the P4HB immunostaining and the H&E histological staining. MC coordinated in vitro study. TL coordinated MS analysis. LH initiated the study and drafted the manuscript. All authors read and approved the final manuscript.

## Supplementary Material

Additional file 1 **A list of proteins identified from an isolated fraction of PSI-induced inclusions in PC12 cells by MALDI-TOF MS PMF**. MS data were specifically searched for proteins by proteomic analysis. Click here for file

Additional file 2 **MS spectra of the unknown proteins of interest**. The eight proteins were incompletely determined by the Profound search engine using the single NCBInr database alone. Click here for file

Additional file 3 **A list of the identified chaperone proteins with both putative similarities between protein families and predictable resources for subcellular localization**. A portion of seventeen chaperone proteins have been found in available literatures to share some similarity between protein families and to specialize in various cellular localization resources. Click here for file
