# Supplementary material for: Proteomic characterization of an isolated fraction of synthetic proteasome inhibitor (PSI)-induced inclusions in PC12 cells might offer clues to aggresomes as a cellular defensive response against proteasome inhibition by PSI
Source: BMC Neurosci. 2010 Aug 12;11:95. doi: 10.1186/1471-2202-11-95 (PMC2928238; doi:10.1186/1471-2202-11-95)
Supplement: Additional file 1 — A list of proteins identified from an isolated fraction of PSI-induced inclusions in PC12 cells by MALDI-TOF MS PMF. MS data were specifically searched for proteins by proteomic analysis. [file 1471-2202-11-95-S1.DOC]

Additional file 1. A list of proteins identified from an isolated fraction of PSI-induced inclusions in PC12 cells by MALDI-TOF MS PMF

| Protein No. and Accession No.  and functional categories | Expectation | Coverage | Theoretic pI | Theoretic mass | Measured peptides | Matched peptides | Probability-based Mowse score | | | | | | Protein name and abbreviation |
| --- | --- | --- | --- | --- | --- | --- | --- | --- | --- | --- | --- | --- | --- |
|  |  |  |  |  |  |  | NCBInr data base SwissProt data base MSDB data base | | | | | |  |
|  |  |  |  |  |  |  | Top score Runner up Top score Runner up Top score Runner up | | | | | |  |
| 1 gi|3319077|pbd|1TOH | 0.000 | 47.5 | 5.5 | 39.60 | 20 | 12 | 119 | 40 | 105 | 38 | 105 | 38 | Tyrosine hydroxylase (TH) |
| 2 gi|13928824|ref|NP_113791.1 | 0.014 | 25.5 | 4.6 | 29.27 | 11 | 6 | 82 | 49 | 80 | 48 | 82 | 34 | 14-3-3epsilon (or tyrosine3-monoxygenase activation protein epsilon) |
| I Synaptic function and neurotransmission | | | | | | | | | | | | | |
| 3 gi|1661221|gb|AAB18412.1 | 0.001 | 28.4 | 4.8 | 28.30 | 11 | 6 | 100 | 39 | 100 | 39 | 100 | 39 | cAMP-dependent protein kinase type I regulatory subunit (PKA-RI) |
| 4 gi|2130649|gb|AAD05363.1 | 0.015 | 20.9 | 5.8 | 35 | 21 | 8 | 75 | 33 | 75 | 40 | 75 | 33 | Eukaryotic elongation factor 2 (eEF-2) |
| 5 gi|62641108|ref|XP_215037.3 | 0.001 | 25.8 | 5.2 | 38.09 | 13 | 7 | 67 | 40 | — | 38 | — | 40 | Eukaryotic translation initiation factor 3 subunit epsilon (eIF3epsilon) |
| 6 gi|8393693|reflNP_058834.1 | 0.003 | 20.0 | 4.8 | 32.92 | 6 | 4 | 65 | — | 64 | — | 64 | — | Laminin receptor 1 (LR1; or 40S ribosomal protein SA) |
| 7 gi|62641274|ref|XP_215069.3 | 0.000 | 31.3 | 8.8 | 56.63 | 14 | 12 | 147 | 40 | 141 | 38 | — | 39 | Tu translation elongation factor, mitochondrial (EF-Tumt) |
| II Gene transcription | | | | | | | | | | | | | |
| 8 gi|50927313|gb|AAH78829.1 | 0.000 | 39.2 | 4.9 | 50.83 | 19 | 15 | 112 | 50 | 112 | 40 | 112 | 40 | Tubulin alpha1 (or tubulin alpha 6) |
| 9 gi|38014544|gb|AAH60540.1 | 0.001 | 25.0 | 4.9 | 24.42 | 8 | 5 | 79 | 52 | 79 | 24 | 79 | 52 | Tubulin beta 5 (tubb5; or tubulin beta 15) |
| III Protein trafficking | | | | | | | | | | | | | |
| 10 gi|6978491|reflNP_036630.1 | 0.000 | 33.2 | 6.3 | 36.24 | 11 | 9 | 63 | 44 | 63 | 44 | 63 | 44 | Aldehyde reductase 1 (AR1) |
| 11 gi|203476|gb|AAA40933.1 | 0.000 | 34.6 | 5.4 | 45.57 | 12 | 9 | 88 | 41 | 86 | 27 | 88 | 37 | Creatine kinase B (CKB) |
| 12 gi|50926833|gblAAH78896.1 | 0.000 | 30.0 | 6.2 | 47.45 | 12 | 10 | 127 | 53 | 127 | 27 | 127 | 33 | Enolase 1 (Eno1; or 2-phospho-D-glycerate hydrolyase) |
| 13 gi|38566122|gb|AAH62226.1 | 0.002 | 20.2 | 8.6 | 36.50 | 8 | 5 | 70 | 31 | 70 | 21 | 70 | 22 | Enoyl coenzyme A hydratase 1 (Enoyl-Co A hydratase) |
| 14 gi|6980956|reflNP-036702.1 | 0.000 | 22 | 8.3 | 61.74 | 12 | 9 | 100 | 43 | 100 | 31 | 100 | 39 | Glutamate dehydrogenase1 (GDH1) |
| 15 gi|16758446|reflNP-446090.1 | 0.004 | 18.6 * | 6.5 | 40.05 | 6 | 5 | 76 | 58 | 75 | 24 | 75 | 24 | NAD(+)-dependent isocitrate dehydrogenase (NAD-specific ICDH) |
| 16 gi|38181543|gblAAah61541.4 | 0.000 | 39.6 | 8.4 | 53.49 | 18 | 15 | 123 | 43 | 186 | 34 | 122 | 38 | Pyruvate kinase M2 (Pkm2) |
| 17 gi|8248819|gblAAB19888.2 | 0.002 | 42.5 | 7.1 | 28.94 | 21 | 7 | 70 | 41 | 70 | 26 | 70 | 26 | Phosphoglycerate mutase type B subunit (PGM-B) |
| 18 gi|34852560|reflXP-215416.2 | 0.001 | 38.4 | 5.3 | 33.21 | 25 | 9 | 76 | 35 | — | 33 | 70 | 33 | Pyrophosphatase (PPase) |
| 19 gi|38541404|gblAAH61999.1 | 0.000 | 19.0 * | 8.2 | 86.16 | 18 | 11 | 86 | 42 | 86 | 34 | 86 | 35 | Aconitase 2, mitochondrial (Aco2) |
| 20 gi|1374715|gb|AAB02288.1 | 0.000 | 45.1 | 4.9 | 51.18 | 20 | 15 | 197 | 44 | 191 | 44 | 197 | 44 | ATP synthase beta subunit (F1-ATPase beta) |
| 21 gi|51948476|reflP-0010042501 | 0.000 | 29.0 | 5.6 | 63.32 | 15 | 11 | 79 | 50 | 79 | 35 | 79 | 41 | Ubiquinol-cytochrome c reductase core protein 1 (Uqcrc1) |
| IV Mitochondrial function and metabolism | | | | | | | | | | | | | |
| 22 gi|207012|gb|AAA42160.1 | 0.000 | 31.6 | 5.9 | 15.86 | 5 | 4 | 79 | — | 78 | — | 79 | — | Cu, Zn superoxide dismutase (C, Z-SOD) |
| V Antioxidant defense mechanisms | | | | | | | | | | | | | |
| 23 gi|9247201|gblAAB31934.2 | 0.000 | 42.5 | 8.3 | 39.03 | 34 | 13 | 135 | 57 | 135 | 42 | 137 | 43 | Annexin II (or Lipocortin II) |
| 24 gi|2981437|gb|AAC06290.1 | 0.000 | 47.4 | 5.0 | 33.95 | 14 | 12 | 173 | 56 | 171 | 47 | 173 | 47 | Annexin V (or Lipocortin V) |
| 25 gi|37999910|sp|P55260|ANXA4 | 0.007 | 20.4 | 5.3 | 36.17 | 7 | 6 | 71 | 45 | 70 | 42 | 71 | 41 | Zymogen granule membrane associated protein (ZAP36; or Annexin IV) |
| VI Apoptotic signal transduction | | | | | | | | | | | | | |
| 26 gi|62661453|reflXP_341315.2 | 0.000 | 18.4 * | 9.4 | 37.40 | 9 | 6 | 84 | 31 | 80 | 23 | 84 | 31 | Proteasome subunit beta 5 (PSMB5) |
| 27 gi|38181888|gb|AAH61542.1 | 0.000 | 30.7 | 5.7 | 49.00 | 18 | 13 | 79 | 42 | 79 | 42 | 79 | 42 | Proteasome 26S subunit ATPase 2 (PSMC2; or MSS1) |
| 28 gi|11265288|pir|T43799 | 0.000 | 32.4 | 8.9 | 42.18 | 14 | 11 | 88 | 37 | 87 | 21 | 88 | 32 | Proteasome 26S subunit ATPase 5 (PSMC5; or p45) |
| 29 gi|34869622|ref|XP_214147.2 | 0.002 | 23.3 | 7.8 | 46.06 | 26 | 8 | 70 | 37 | 70 | 21 | 70 | 22 | Proteasome 26S subunit ATPase 6 (PSMC6; or p42) |
| 30 gi|34872897|ref|XP_220754.2 | 0.001 | 21.6 | 8.7 | 54.40 | 11 | 8 | 122 | 42 | 116 | 32 | 112 | 42 | Proteasome 26S subunit non-ATPase 11 (PSMD11; or p44.5) |
| 31 gi|62641588|ref|XP_1344977.2 | 0.000 | 38.8 | 5.5 | 43.08 | 22 | 12 | 173 | 56 | 171 | 47 | 173 | 47 | Proteasome 26S subunit non-ATPase 13 (PSMD13; or p40.5) |
| VII Ubiquitin dependent protein degradation | | | | | | | | | | | | | |
| 32 gi|14010865|re|NP_114176.1 | 0.006 | 33.7 | 6.1 | 22.8 | 15 | 6 | 67 | 38 | 67 | 26 | 67 | 29 | 27-kD heat shock protein 1 (HSP27) |
| 33 gi|7767105|pdb|1DVGIB | 0.003 | 28.1 | 6.0 | 29.89 | 11 | 5 | 69 | 30 | 69 | 33 | 69 | 35 | Heat shock protein 32 (HSP32; or heme oxygenase-1) |
| 34 gi|38382858|gb|AAH62393.1 | 0.000 | 38.0 | 5.9 | 57.06 | 23 | 16 | 198 | 47 | 198 | 30 | 198 | 37 | 58-kD glucose regulated protein (GRP58; or ERp57) |
| 35 gi|415898|emb|CAA81642.1 | 0.000 | 34.5 | 5.6 | 50.50 | 16 | 11 | 180 | 40 | 180 | 29 | 180 | 33 | 70-kD heat shock protein 1A/1B (HSP70) |
| 36 gi|56385|emb|CCA49670.1 | 0.000 | 39.5 | 5.4 | 71.14 | 24 | 20 | 169 | 44 | 169 | 39 | 169 | 39 | 71-kD heat shock cognate protein (HSC70) |
| 37 gi|55584140|s|P48721 | 0.000 | 25.9 | 6.0 | 74.11 | 16 | 13 | 163 | 48 | 163 | 37 | 163 | 37 | 75-kD glucose regulated protein (GRP75) |
| 38 gi|58865372|ref|NP_001011901.1 | 0.000 | 22.4 | 5.4 | 97.37 | 17 | 15 | 70 | 40 | 70 | 39 | 70 | 39 | 105-kD heat shock protein 1 (HSP105) |
| 39 gi|10720174|sp|Q63617|OXRP | 0.000 | 19.5 * | 5.1 | 111.5 | 17 | 16 | 121 | 52 | 121 | 44 | 121 | 44 | 150-kD oxygen regulated protein (ORP150) |
| 40 gi|488838|emb|CAA55891.1 | 0.000 | 22.5 | 4.9 | 47.60 | 13 | 7 | 90 | 42 | 89 | 31 | 90 | 32 | Calcium-binding protein 1 (CaBP1) |
| 41 gi|2511701|emblCAA05100.1 | 0.001 | 23.2 | 4.4 | 37.09 | 7 | 5 | — | 57 | 57 | 31 | 57 | 37 | CBP-50 protein ( or Crocalbin) |
| 42 gi|1334284|emb|CAA 37654.1 | 0.000 | 46.3 | 5.3 | 58.08 | 21 | 17 | 113 | 34 | 109 | 41 | 113 | 41 | Mitochondrial heat shock protein 60 (HSP60) |
| 43 gi|1051270|gb|AAA80544.1 | 0.008 | 41.2 | 4.7 | 27.95 | 33 | 8 | 74 | 33 | 74 | 22 | 74 | 29 | Protein kinase C inhibitor protein (KCIP-1; or 14-3-3zeta) |
| 44 gi|38197382|gb|AAH61857.1 | 0.000 | 28.7 | 4.8 | 57.33 | 21 | 11 | 71 | 38 | 71 | 37 | 71 | 38 | Prolyl-4-hydroxylase beta polypeptide (P4HB) |
| 45 gi|38181876|gblAAH61529.1 | 0.001 | 18.2 * | 6.4 | 63.18 | 12 | 9 | 87 | 45 | 87 | 41 | 87 | 41 | Stress-induced-phosphoprotein 1(STI1; or Hop; or P60) |
| 46 gi|54400730|reflNP-001005905.1 | 0.000 | 26.0 | 6.0 | 57.78 | 12 | 10 | 100 | 33 | 100 | 31 | 100 | 37 | T-complex polypeptide 1 beta subunit (TCP-1beta) |
| 47 gi|51260037|gblAAH79441.1 | 0.000 | 24.0 | 5.5 | 59.98 | 15 | 10 | 53 | — | 53 | 39 | 53 | — | T-complex polypeptide 1 epsilon subunit (TCP-1epsilon) |
| 48 gi|38014694|gb|AAH60518.1 | 0.000 | 30.1 | 5.1 | 90.02 | 20 | 15 | 109 | 42 | 109 | 35 | 109 | 35 | Valosin-containing protein (VCP) |
| VIII Protein folding and transport | | | | | | | | | | | | | |
| 49 gi|50925725|gb|AAH7917.1 | 0.005 | 18.4 * | 6.2 | 39.32 | 9 | 4 |  |  |  |  |  |  |  |
| 50 gi|62643335|ref|XP-579983.1 | 0.033 | 19.4 * | 8.01 | 20.62 | 22 | 3 |  |  |  |  |  |  |  |
| 51 gi|18426844|ref|569100.1 | 0.000 | 22.0 | 5.9 | 50.35 | 14 | 9 |  |  |  |  |  |  |  |
| 52 gi|50657380|ref|NP-001002807.1 | 0.003 | 45.6 | 5.1 | 27.31 | 29 | 7 |  |  |  |  |  |  |  |
| 53 gi|11560095|ref|NP071609.1 | 0.045 | 17.4 * | 6.6 | 54.55 | 21 | 5 |  |  |  |  |  |  |  |
| 54 gi|62651904|reflXP_576206.1 | 0.000 | 19.7 | 4.7 | 73.04 | 21 | 18 |  |  |  |  |  |  |  |
| 55 gi|37361854|gblAAQ91040.1 | 0.000 | 26.3 | 5.9 | 40.76 | 10 | 8 |  |  |  |  |  |  |  |
| 56 gi|34872057|reflXP_213765.2 | 0.011 | 22.4 | 6.6 | 58.46 | 15 | 8 |  |  |  |  |  |  |  |
| IX Unknown | | | | | | | | | | | | | |

Expectation: a chance of incorrect identification. Coverage: a ratio of the protein sequence covered by matched peptides. Accession No., protein database entry of a protein candidate in the single NCBInr database. “—”: no values of top score or runner up for candidate proteins in the analysis of probability-based Mowse score.

*: In some cases where conventional MALDI systems typically provide sequence coverage of up close to 20% for low level standard protein digests, protein identification was not affected by experimental mass value matching in a search of NCBI database with ProFound search engine (<http://prowl.rockefeller.edu/prowl-cgi/profound.exe>).
