# Supplementary material for: Proteomic characterization of an isolated fraction of synthetic proteasome inhibitor (PSI)-induced inclusions in PC12 cells might offer clues to aggresomes as a cellular defensive response against proteasome inhibition by PSI
Source: BMC Neurosci. 2010 Aug 12;11:95. doi: 10.1186/1471-2202-11-95 (PMC2928238; doi:10.1186/1471-2202-11-95)
Supplement: Additional file 3 — A list of the identified chaperone proteins with both putative similarities between protein families and predictable resources for subcellular localization. A portion of seventeen chaperone proteins have been found in available literatures to share some similarity between protein families and to specialize in various cellular localization resources. [file 1471-2202-11-95-S3.DOC]

Additional file 3. A list of a profile of chaperone proteins with both putative similarities between protein families and predictable resources for subcellular localization.

Continued

| Protein family | Cellular localization resource | | |  | Protein family | Cellular localization resource | | |
| --- | --- | --- | --- | --- | --- | --- | --- | --- |
|  | Cytoplasm | Endoplasmic reticulum lumen | Mitochondria matrix |  |  | Cytoplasm | Endoplasmic reticulum lumen | Mitochondria matrix |
| The 14-3-3 family | | | |  | 35 / HSP70 | **+** |  |  |
| 43 / 14-3-3zeta | **+** |  |  |  | 36 / HSC70 | **+** |  |  |
| The acronym Ca2þ-binding protein of 45 kD, Reticulocalbin, ER Ca2þ-binding protein of 55 kD, and Calumenin family | | | |  | 37 / GRP75 |  |  | **+** |
| 41 / CBP-50 protein |  | **+** |  |  | 38 / HSP105 | **+** |  |  |
| The chaperone cofactors | | | |  | 39 / ORP150 |  | **+** |  |
| 45 / STI1 | **+** |  |  |  | The small heat shock proteins (sHSP) family | | | |
| The chaperonin family | | | |  | 32 / HSP27 | **+** |  |  |
| 42 / HSP60 |  |  | **+** |  | 33 / HSP32 |  | **+** |  |
| 46 / TCP-1 beta | **+** |  |  |  | The protein disulfide isomerase (PDI) family | | | |
| 47 / TCP-1epsilon | **+** |  |  |  | 34 / GRP58 |  | **+** |  |
| The family of ATPases associated with various cellular activities | | | |  | 40 / CaBP1 |  | **+** |  |
| 48 / VCP | **+** |  |  |  | 44 / P4HB |  | **+** |  |
| The heat shock protein 70 (HSP70) family | | | |  |  |  |  |  |

**+:** A chaperone protein belonging to a protein family and obtaining information on a subcellular localization resource are indicated as a cross sign.
